# Optimal Scheduling of Energy Systems for Gas-to-Methanol Processes Using Operating Zone Models and Entropy Weights

**DOI:** 10.3390/e27030324

**Published:** 2025-03-20

**Authors:** Xueteng Wang, Mengyao Wei, Jiandong Wang, Yang Yue

**Affiliations:** 1 College of Electrical Engineering and Automation, Shandong University of Science and Technology, Qingdao 266590, China; wxtskd2@163.com (X.W.); mywei9701@163.com (M.W.); 2 Shandong Rongxin Group Co., Ltd., Zoucheng 273517, China; rxmhyy@163.com

**Keywords:** gas-to-methanol process, operating zone model, entropy weight, optimal scheduling, visual manner

## Abstract

In coal chemical industries, the optimal allocation of gas and steam is crucial for enhancing production efficiency and maximizing economic returns. This paper proposes an optimal scheduling method using operating zone models and entropy weights for an energy system in a gas-to-methanol process. The first step is to develop mechanistic models for the main facilities in methanol production, namely desulfurization, air separation, syngas compressors, and steam boilers. A genetic algorithm is employed to estimate the unknown parameters of the models. These models are grounded in physical mechanisms such as energy conservation, mass conservation, and thermodynamic laws. A multi-objective optimization problem is formulated, with the objectives of minimizing gas loss, steam loss, and operating costs. The required operating constraints include equipment capacities, energy balance, and energy coupling relationships. The entropy weights are then employed to convert this problem into a single-objective optimization problem. The second step is to solve the optimization problem based on an operating zone model, which describes a high-dimensional geometric space consisting of all steady-state data points that satisfy the operation constraints. By projecting the operating zone model on the decision variable plane, an optimal scheduling solution is obtained in a visual manner with contour lines and auxiliary lines. Case studies based on Aspen Hysys are used to support and validate the effectiveness of the proposed method.

## 1. Introduction

A gas-to-methanol process comprises primarily the preparation and purification of the feedstock gas, as well as the synthesis and refining of methanol [1,2]. The process entails the supply, demand, conversion, and storage of various energy media, mainly including gas and steam. The irregular rhythm of methanol production often leads to serious energy waste problems. It is, therefore, necessary to implement scientific and reliable optimization schemes to ensure the safety and economic viability of the integrated gas and steam energy system for the gas-to-methanol process [3,4,5].

In recent years, optimal scheduling for energy systems has received increasing attention from both industry and academic communities [6,7,8,9,10]. Abdollahi et al. [11] conducted multi-objective optimization for the design of a small, distributed combined cooling, heating, and power system, incorporating reliability and availability into the thermo-economic model and using risk analysis for final decision making. Di Somma et al. [12] obtained a mixed-integer linear programming (MILP) model for a building energy supply system, focusing on energy costs and efficiency under the combination of fossil and renewable energy. Zeng et al. [13] developed a multi-period MILP model for complex, large-scale energy optimization problems, targeting the actual operating characteristics of various energy-coupling equipment in iron and steel plants. Daraei et al. [14] assessed the impact of renewable energy systems on the production planning of local combined heat and power plants, validating the feasibility of the optimization model through extreme operating conditions of energy supply and demand. Wei et al. [15] proposed an optimization scheduling model for a multi-period gas–steam–electricity coupled energy system, considering gas holder penalties, energy procurement costs, and carbon emission costs. Zhou et al. [16] established a two-layer optimization scheduling model for the operation feasibility of gas turbine standby in multi-energy system scheduling. Alhumaid et al. [17] built a mixed-integer nonlinear programming model based on minimizing the total operational costs of energy storage systems by evaluating combinations of energy storage technologies and renewable energy sources. Wirtz et al. [18] analyzed 24 MILP models for multi-sector energy system design and found that incorporating part-load efficiencies minimizes system costs. Guo et al. [19] optimized integrated electric and thermal energy systems with economics and carbon emissions and adopted a multi-objective quantity regulation scheduling method. Lamari et al. [20] introduced a multi-objective particle swarm optimization algorithm to reduce emissions and operating costs in virtual power plant systems. Monfaredi et al. [21] studied a novel optimal operation and planning method for integrated energy systems (IESs) using an adaptive particle swarm optimization technique. Abu-Rayash et al. [22] designed an integrated solar and wind energy system with compressed air and battery storage, achieving high efficiency and low emissions for a small city. Chen et al. [23] presented an exergy analysis model and joint solution framework for IESs, providing accurate optimization and evaluation. Liu et al. [24] formulated a day-ahead scheduling method for regional IESs with hot dry rock, reducing system costs, energy consumption, and emissions. There are two main limitations in the existing literature. First, these studies do not cover the energy system for the gas-to-methanol process. Second, a visual decision-making method for the multi-objective optimization of this process is not available.

The main contribution of this paper is to propose an optimal scheduling method using operating zone models and entropy weights for an energy system in a gas-to-methanol process. First, mechanistic models are developed based on the physical mechanisms of major production facilities, and unknown model parameters are estimated based on historical data through the genetic algorithm. A multi-objective optimization problem is formulated, with the objectives of minimizing gas loss, steam loss, and operating costs. The required operating constraints include equipment capacities, energy balance, and energy coupling relationships. The entropy weight approach is then employed to convert this problem into a single-objective optimization problem. Second, the optimization problem is solved based on an operating zone model, as it is composed of all steady-state data points from the mechanistic models satisfying all operation constraints, to formulate a high-dimensional geometric space. By projecting the operating zone model on the decision variable plane, an optimal scheduling solution is obtained in a visual manner with contour lines and auxiliary lines. In real industrial production, the gas-to-methanol process often relies on experience-based scheduling, leading to a lack of trust among industrial plant operators in optimizing-based decision-making. The main challenge is how to develop an optimization model for the gas-to-methanol process and to facilitate its visual decision-making within a multi-objective framework. To the best of our knowledge, such an optimal scheduling method using operating zone models and entropy weights for an energy system in a gas-to-methanol process is the first one in the literature. The proposed method resolves the above-mentioned challenge.

The remainder of this paper is structured as follows. Section 2 introduces the problem description. Section 3 covers mechanistic modeling and optimization problem formulation. Section 4 presents detailed steps of the proposed method. Case studies based on Aspen Hysys are provided in Section 5. Concluding remarks are given in Section 6.

## 2. Problem Description

**Figure 1:**
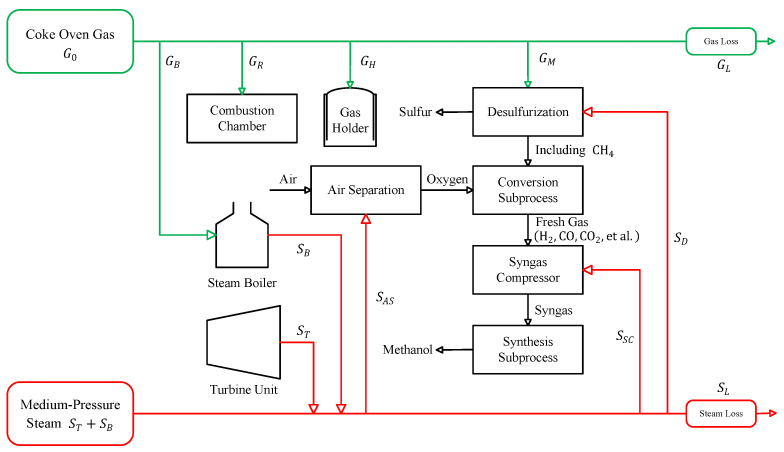
Schematic diagram of the gas-to-methanol process.

A schematic diagram of an energy system is shown in Figure 1, consisting of a gas subsystem (green) and a steam subsystem (red) in a gas-to-methanol process. In the gas subsystem, the coke oven is the only gas production unit. Coal is distilled in the coke oven to produce the crude gas, which is purified through wet coking and is treated to obtain the gas $G_{0}$. There are four consumption branches of $G_{0}$: recycled gas, $G_{R}$, for the combustion chamber, combustion gas, $G_{B}$, for the steam boiler, conversion gas, $G_{M}$, for the methanol production, and storage gas, $G_{H}$, for the gas holder. Here, $G_{R}$ is maintained in a certain ratio to $G_{0}$ to provide the heat required for coking.

In the steam subsystem, the boiler and turbine unit are the facilities for producing steam. The boiler uses the heat of gas, $G_{B}$, combustion to convert deaerated water into steam, $S_{B}$. The steam, $S_{T}$, from the turbine unit is extracted from the medium-pressure cylinder after cooling and pressure reduction. The steam-consuming facilities are mainly concentrated on methanol production. Organic sulfur is first removed from the gas, $G_{M}$, via desulfurization so that it can enter the conversion subprocess. In the converter, ${\mathrm{CH}}_{4}$ and unsaturated hydrocarbons undergo oxidation and steam conversion reactions with pure oxygen to produce $H_{2}$, CO, ${\mathrm{CO}}_{2}$, and other active components required for the synthesis of methanol. These components are then transferred to the synthesis subprocess via a syngas compressor, and methanol is synthesized in the presence of a catalyst. Here, the steam required to convert the gas, $G_{M}$, includes steam, $S_{D}$, consumed via desulfurization, steam, $S_{AS}$, consumed via air separation to provide pure oxygen for the conversion subprocess, and steam, $S_{SC}$, consumed via the syngas compressor.

Due to the fluctuations in methanol production, there are often imbalances between the production and consumption of the gas and steam subsystems. These imbalances are reflected in the gas loss, $G_{L}$, and steam loss, $S_{L}$. Although the gas holder can buffer the imbalance in the gas subsystem, the small volume constraint of the gas holder often leads to a temporary excess or shortage of gas. In the steam subsystem, due to the lack of effective storage methods, industrial plant operators need to produce excess steam as a backup in practice to ensure the safety of methanol production and the efficient utilization of gas.

Given the current values of the total gas flow, $G_{0}$, steam extraction, $S_{T}$, and gas holder volume, $V_{H}$, our objective is to solve an optimization problem in order to minimize the gas loss, steam loss, and operating costs. Operation constraints are mainly from equipment capacities, energy balance, and energy coupling relationships. The optimal solutions of the gases $G_{M}$, $G_{B}$, and $G_{H}$ need to be determined, preferably in a visual manner.

## 3. Modeling and Optimization Problem

This section establishes the mechanistic models in Section 3.1 and formulates the optimization problem in Section 3.2.

### 3.1. Mechanistic Modeling

This subsection presents the mechanistic modeling for desulfurization, air separation, the syngas compressor, and the steam boiler.

*(1) Desulfurization modeling.*

The molten sulfur kettle utilized in desulfurization is a shell-and-tube heat exchanger and serves as the primary steam-consuming facility. The heat-balance relationship of the steam and sulfur is derived from the first law of thermodynamics as

(1) $$Q_{su}=Q_{st},$$

The energy exchange between the heat $Q_{su}$, absorbed via the sulfur, and the heat $Q_{st}$, released via the steam, is calculated as

(2) $$\begin{array}{cc} \; & Q_{su}=\left(T_{su,o}-T_{su,i}\right)C_{su}F_{su},\\ \; & Q_{st}=\left(T_{st,i}-T_{st,o}\right)C_{st}S_{D}. \end{array}$$

According to the operating manual, there is a ratio between the sulfur flow, $F_{su}$, and the gas flow, $G_{M}$, such that $F_{su}=\alpha_{D}G_{M}+\beta_{D}$, where $\alpha_{D}$ is a ratio factor, and $\beta_{D}$ is a correction factor.

The mechanistic model of desulfurization can be obtained according to (1) and (2) as

(3) $$S_{D}=\frac{T_{su,o}-T_{su,i}}{T_{st,i}-T_{st,o}}\left(\theta_{D,1}G_{M}+\theta_{D,2}\right),$$

where $\theta_{D,1}=\frac{C_{su}\alpha_{D}}{C_{st}}$ and $\theta_{D,2}=\frac{C_{su}\beta_{D}}{C_{st}}$ are the unknown parameters of the model [25].

*(2) Air separation modeling.*

The turbine serves as the primary facility for steam consumption in air separation. It follows a one-to-two configuration, where one turbine drives one air compressor and one booster compressor.

First, because of the identical operational principles shared by the two compressors, a unified mechanistic model is taken here. The power consumption, $W_{C,s}$, of the *s*-th compressor is calculated as

(4) $$W_{C,s}=\frac{RT_{C,i,s}ln\frac{P_{C,o,s}}{P_{C,i,s}}\cdot \rho_{A,s}F_{A}}{3600\eta_{I,s}},s=1,2.$$

Here, the inlet air flow, $F_{A}$, can be calculated from the material and nitrogen component balances in the distillation tower as

$$F_{A}=r_{O}G_{M}\cdot \frac{X_{O}-X_{N}}{X_{A}-X_{N}},$$

where $r_{O}$ is the appropriate ratio of oxygen to the gas $G_{M}$ to ensure the normal progress of oxidation and steam conversion reactions.

Next, the turbine is assumed to be an isentropic process. The turbine power output is determined by the decrease in steam energy as it passes through the turbine. This can be derived from the energy equation for adiabatic expansion as

(5) $$W_{T1}=\frac{\eta_{T1}S_{AS}C_{st}\left(T_{st,i}-T_{T1,o}\right)}{3600}.$$

According to the ideal gas law, as well as the relationship between pressure and temperature, the turbine exhaust temperature $T_{T1,o}$ is determined as

(6) $$T_{T1,o}=T_{st,i}{\left(\frac{P_{T1,o}}{P_{st,i}}\right)}^{\frac{k_{T1}-1}{k_{T1}}}.$$

Last, based on the principle of power conservation, i.e., $W_{C,1}+W_{C,2}=W_{T1}$, the mechanistic model for the air separation is calculated from (4)–(6) as

(7) $$S_{AS}=\frac{\left(T_{C,i,1}ln\frac{P_{C,o,1}}{P_{C,i,1}}\cdot \theta_{AS,1}+T_{C,i,2}ln\frac{P_{C,o,2}}{P_{C,i,2}}\cdot \theta_{AS,2}\right)G_{M}}{T_{st,i}\left(1-{\left(\frac{P_{T1,o}}{P_{st,i}}\right)}^{\frac{k_{T1}-1}{k_{T1}}}\right)}+\theta_{AS,3}.$$

Here, $\theta_{AS,1}=\frac{R\rho_{A,1}r_{O}\cdot \frac{X_{O}-X_{N}}{X_{A}-X_{N}}}{{\eta_{T1}C_{st}\eta }_{I,1}}$, $\theta_{AS,2}=\frac{R\rho_{A,2}r_{O}\cdot \frac{X_{O}-X_{N}}{X_{A}-X_{N}}}{{\eta_{T1}C_{st}\eta }_{I,2}}$, and $\theta_{AS,3}$ are the unknown parameters of the model [26]. The ideal gas model is an approximate assumption for the real gas behavior. The parameter $\theta_{AS,3}$ is introduced to mitigate the error caused by this assumption in the air separation model. A similar approach was used in the literature to establish a steam turbine model [27].

*(3) Syngas Compressor Modeling*

The syngas compressor adopts three-stage compression, and its modeling logic is similar to that of the air separation. The power consumption $W_{SC,s}$ for the *s*-th stage of the syngas compressor can be expressed as

(8) $$W_{SC,s}=\frac{\frac{m_{s}}{m_{s}-1}\rho_{SC,s}\frac{R_{u}}{M_{s}}T_{SC,i,s}F_{s}\left({\left(\frac{P_{SC,o,s}}{P_{SC,i,s}}\right)}^{\frac{m_{s}-1}{m_{s}}}-1\right)}{3600\eta_{P,s}},s=1,2,3.$$

Here, $F_{1}$ and $F_{2}$ represent the fresh gas, with their empirical ratios to $G_{M}$ expressed as $F_{1}=F_{2}=r_{f}G_{M}$. $F_{3}$ denotes the synthesis gas formed through the mixture of fresh gas and cycle gas, i.e., $F_{3}=r_{f}G_{M}+f\left(G_{M}\right)$, where $f\left(G_{M}\right)=C_{1}{\left(G_{M}\right)}^{2}+C_{2}G_{M}+C_{3}$ is a coupling relationship between the cycle gas and $G_{M}$. The polytropic index $m_{s}$ of the *s*-th compressor in (8) can be calculated as

(9) $$m_{s}=\frac{\ln\left(\frac{P_{SC,o,s}}{P_{SC,i,s}}\right)}{\ln\left(\frac{T_{SC,i,s}}{T_{SC,o,s}}\right)+\ln\left(\frac{P_{SC,o,s}}{P_{SC,i,s}}\right)}.$$

The formula for the turbine output power in (5) can be rewritten as

(10) $$W_{T2}=\frac{\eta_{T2}S_{SC}C_{st}T_{st,i}\left(1-{\left(\frac{P_{T2,o}}{P_{st,i}}\right)}^{\frac{k_{T2}-1}{k_{T2}}}\right)}{3600}.$$

According to power conservation, i.e., $\sum_{s=1}^{3}W_{SC,s}=W_{T2}$, the mechanistic model of the syngas compressor can be derived from (8)–(10) as

(11) $$S_{SC}=\frac{\sum_{s=1}^{3}\theta_{SC,s}\frac{\ln\left(P_{SC,o,s}\right)-\ln\left(P_{SC,i,s}\right)}{\ln\left(T_{SC,o,s}\right)-\ln\left(T_{SC,i,s}\right)}T_{SC,i,s}F_{s}\cdot {\left(\frac{P_{SC,o,s}}{P_{SC,i,s}}\right)}^{\frac{\ln\left(T_{SC,o,s}\right)-\ln\left(T_{SC,i,s}\right)}{\ln\left(P_{SC,o,s}\right)-\ln\left(P_{SC,i,s}\right)}}-1}{T_{st,i}\left(1-{\left(\frac{P_{T2,o}}{P_{st,i}}\right)}^{\frac{k_{T2}-1}{k_{T2}}}\right)}+\theta_{SC,4},$$

where $\theta_{SC,s}=\frac{\rho_{SC,s}\frac{R_{u}}{M_{s}}}{\eta_{P,s}\eta_{T2}C_{st}}$ and $\theta_{SC,4}$ are the unknown parameters of the model [28].

*(4) Steam boiler modeling.*

The steam boiler transforms the chemical energy of combusted gas into thermal energy to generate steam. The heat balance equation is

(12) $$q_{1}+q_{2}+q_{3}+q_{4}=100\%,$$

where the effective utilization rate, $q_{1}$, for the boiler is found to be

(13) $$q_{1}=\frac{100S_{B}\left(H_{S}-H_{FW}\right)}{Q_{B}G_{B}}.$$

The exhaust loss $q_{2}$ is one of the major losses in the boiler, and it can be expressed using an empirical formula as

(14) $$q_{2}=\left(k_{B,1}+k_{B,2}\alpha_{EG}\right)\cdot \left(1-\frac{q_{4}}{100}\right)\cdot \frac{T_{EG}-T_{CA}}{100}.$$

Here, $q_{3}$ is the heat loss due to the incomplete combustion of gases, and $q_{4}$ is the heat loss due to the incomplete combustion of solid fuels. These losses are relatively minor in the overall boiler-heat balance. Therefore, they are treated as unknown parameters in the model.

The mechanistic model for the steam boiler is developed from (12)–(13) as

(15) $$S_{B}=\left(\theta_{B,1}-\theta_{B,2}\left(T_{EG}-T_{CA}\right)\right)G_{B},$$

where the unknown model parameters are [29]

$$\begin{array}{cc} \; & \theta_{B,1}=\frac{\left(1-q_{3}-q_{4}\right)Q_{B}}{100(H_{S}-H_{FW})},\\ & \theta_{B,2}=\frac{\left(k_{B,1}+k_{B,2}\alpha_{EG}\right)\cdot \left(1-\frac{q_{4}}{100}\right)Q_{B}}{10^{4}\cdot \left(H_{S}-H_{FW}\right)}. \end{array}$$

*(5) Identification of mechanistic models.*

Unknown parameters of the above mechanistic models can be estimated from historical data. With the desulfurization model as an example for illustration, the unknown parameter $\theta_{D}$ in (3) is estimated by minimizing the sum of squared errors between the historical data ${\left\{S_{D,d}\right\}}_{d=1}^{N_{D}}$ and the model data ${\hat{S}}_{D}$ as

(16) $${\hat{\theta }}_{D}=\underset{\theta_{D}}{\arg\min}\sum_{d=1}^{N_{D}}{\left(S_{D,d}-{\hat{S}}_{D,d}\left(\theta_{D}\right)\right)}^{2}.$$

The genetic algorithm can be used to solve (16) for the estimation of $\theta_{D}$. The parameter estimation formulas for the mechanistic models in (7), (11) and (15) are similar to those for the desulfurization model in (16). Here, the estimated models are represented as

(17) $$\left\{\begin{array}{c} {\hat{S}}_{D}\left(\theta_{D}\right)=f_{D}\left(x_{D};G_{M};\theta_{D}\right),\\ {\hat{S}}_{AS}\left(\theta_{AS}\right)=f_{AS}\left(x_{AS};G_{M};\theta_{AS}\right),\\ {\hat{S}}_{SC}\left(\theta_{SC}\right)=f_{SC}\left(x_{SC};G_{M};\theta_{SC}\right),\\ {\hat{S}}_{B}\left(\theta_{B}\right)=f_{B}\left(x_{B};G_{B};\theta_{B}\right), \end{array}\right.$$

where the measurable variables are

(18) $$\left\{\begin{array}{c} x_{D}=\left[T_{su,i},T_{su,o},T_{st,i},T_{st,o}\right],\\ x_{AS}=\left[T_{C,i,s},T_{st,i},P_{C,i,s},P_{C,o,s},P_{T1,o},P_{st,i}\right],\\ x_{SC}=\left[T_{SC,i,s},T_{SC,o,s},T_{st,i},P_{SC,i,s},P_{SC,o,s},P_{T2,o},P_{st,i}\right],\\ x_{B}=\left[T_{EG},T_{CA}\right], \end{array}\right.$$

and the unknown parameters are

(19) $$\left\{\begin{array}{c} \theta_{D}=\left[\theta_{D,1},\theta_{D,2}\right],\\ \theta_{AS}=\left[\theta_{AS,1},\theta_{AS,2},\theta_{AS,3}\right],\\ \theta_{SC}=\left[\theta_{SC,1},\theta_{SC,2},\theta_{SC,3},\theta_{SC,4}\right],\\ \theta_{B}=\left[\theta_{B,1},\theta_{B,2}\right]. \end{array}\right.$$

Here, $f_{D}\left(\cdot \right)$ is the desulfurization model in (3), $f_{AS}\left(\cdot \right)$ is the air separation model in (7), $f_{SC}\left(\cdot \right)$ is the syngas compressor model in (11), $f_{B}\left(\cdot \right)$ is the steam boiler model in (15).

### 3.2. Formulation of Optimization Problem

On the basis of process knowledge and practical experience in the operation of industrial facilities, the productivity and consumption rates of gas and steam shall meet their equipment capacity constraints.

(20) $$\begin{array}{cc} \; & G_{0,min}\leq G_{0}\leq G_{0,max},\\ \; & S_{T,min}\leq S_{T}\leq S_{T,max},\\ \; & V_{H,min}\leq V_{H}\leq V_{H,max},\\ \; & G_{M,min}\leq G_{M}\leq G_{M,max},\\ \; & G_{B,min}\leq G_{B}\leq G_{B,max}. \end{array}$$

Here, the subscripts min and max are the minimum and maximum values of variables, respectively.

There are four gas consumption paths for the total gas $G_{0}$, namely the recycled gas, $G_{R}$, the conversion gas, $G_{M}$, the combustion gas, $G_{B}$, and the storage gas, $G_{H}$. The gas loss, $G_{L}$, is expressed as

(21) $$G_{L}=G_{0}-\left(G_{B}+G_{M}+G_{R}+G_{H}\right),$$

where $G_{R}$ is returned to the coke oven combustion chamber in a certain ratio, $r_{R}$, with respect to $G_{0}$ to provide the heat required for coking, i.e.,

(22) $$G_{R}=r_{R}\cdot G_{0}.$$

The gas holder serves primarily as a buffer, with the direction of gas flow into (or out of) the gas holder defined as positive (or negative). The volume, $V_{H}$, of the gas holder reflects the frequent imbalances between gas supply and consumption. When there is an excess or shortage of gas supplied via $G_{0}$, the storage gas, $G_{H}$, in the gas holder is adjusted to ensure the normal operation of the gas subsystem. The gas balance constraint is

(23) $$G_{H,min}-G_{H}\leq G_{L}\leq G_{H,max}-G_{H}.$$

Here, $G_{H,min}$ and $G_{H,max}$ are calculated as

(24) $$\begin{array}{cc} \; & G_{H,min}=V_{H,min}-V_{H},\\ \; & G_{H,max}=V_{H,max}-V_{H}, \end{array}$$

where $\left|G_{H,min}\right|$ is the maximum supply from the gas holder to the gas subsystem, and $G_{H,max}$ is the maximum storage from the gas subsystem to the gas holder.

The heat required for the conversion of the gas $G_{M}$ includes the steam consumption, $S_{D}$, for desulfurization, $S_{AS}$ for air separation, and $S_{SC}$ for the syngas compressor. This heat is supplied by coordinating the steam $S_{B}$, generated from burning gas, $G_{B}$, in the boiler, and the steam $S_{T}$, extracted from the turbine unit. The steam loss, $S_{L}$, can be expressed as

(25) $$\begin{array}{cc} S_{L} & =S_{T}+S_{B}-S_{D}-S_{AS}-S_{SC},\\ \; & =S_{T}+f_{B}\left(G_{B}\right)-f_{D}\left(G_{M}\right)-f_{AS}\left(G_{M}\right)-f_{SC}\left(G_{M}\right). \end{array}$$

Here, $f_{D}$, $f_{AS}$, $f_{SC}$, and $f_{B}$ are mechanistic models, as defined in (17).

Due to the difficulty of storing steam, a portion of the steam output from the boiler and turbine unit is reserved as backup steam and is not used directly in production. The ratio of backup steam to the total steam output is defined as the safety margin, $r_{SM}$. The steam supply and demand sides of the system should be subject to the following inequality constraint:

(26) $$\left(S_{T}+f_{B}\left(G_{B}\right)\right)\cdot \left(1+r_{SM}\right)-f_{D}\left(G_{M}\right)-f_{AS}\left(G_{M}\right)-f_{SC}\left(G_{M}\right)\geq 0.$$

In methanol production, extraction steam and total gas are the primary fuels, and their respective purchase costs must be considered. To optimize the utilization of surplus resources, the objective function also accounts for the revenue generated from the sale of surplus steam in the operating costs. The objective function of the operating costs is

(27) $$Cost=c_{st,T}{\cdot S}_{T}+c_{gas}{\cdot G}_{0}-c_{st,MP}\cdot \left(S_{T}+f_{B}\left(G_{B}\right)-f_{D}\left(G_{M}\right)-f_{AS}\left(G_{M}\right)-f_{SC}\left(G_{M}\right)\right).$$

The optimization problem is summarized as

(28) $$\begin{array}{cc} \; & \min.\;\;\mathrm{Objective}\;\mathrm{functions}\;\;\;\;\;\;\;\;\;\;\;\;\;\;\;\mathrm{Equations}\;(21),\;(25)\;\mathrm{and}\;(27),\\ \; & s.t.\;\;\;\mathrm{Equipment}\;\mathrm{capacities}\;\;\;\;\;\;\;\;\;\;\;\;\;\mathrm{Equation}\;(20),\\ \; & \;\;\;\;\;\;\;\mathrm{Energy}\;\mathrm{balance}\;\;\;\;\;\;\;\;\;\;\;\;\;\;\;\;\;\;\;\mathrm{Equations}\;(23)\;\mathrm{and}\;(26),\\ \; & \;\;\;\;\;\;\;\mathrm{Coupling}\;\mathrm{relationships}\;\;\;\;\;\;\;\;\;\;\;\mathrm{Equation}\;(17). \end{array}$$

Given the current values of the input variables $G_{0}$, $S_{T}$, and $V_{H}$ and the nominal values of the measurable variables $x_{D}$, $x_{AS}$, $x_{SC}$, and $x_{B}$ in (18), the decision variables $G_{M}$, $G_{B}$, and $G_{H}$ are determined by solving the optimization problem, and the corresponding steam, $S_{B}$, $S_{D}$, $S_{AS}$, and $S_{SC}$, is calculated from models in (17).

## 4. The Proposed Method

This section describes the detailed steps of the optimal scheduling method.

### 4.1. Formulation of Operating Zone Model

First, given the equipment capacities of the variables $G_{0}$, $S_{T}$, $V_{H}$, $G_{M}$, and $G_{B}$ in (20), the variable step sizes are chosen according to the sensor measurement resolution. All the variables are traversed in a sufficiently large high-dimensional sample space. The data within the sample space are denoted as $\left\{X\left(n\right)|n\in \left[1,N_{1}\right]\right\}$ with $X\left(n\right):=\left[{G_{0}\left(n\right),S_{T}\left(n\right),V_{H}\left(n\right),G}_{M}\left(n\right),G_{B}\left(n\right),G_{H}\left(n\right)\right]$.

Second, data points in ${\left\{X\left(n\right)\right\}}_{n=1}^{N_{1}}$ satisfying the energy balance in (23) and (26) and the coupling relationships in (17) are selected as another dataset, ${\left\{X_{ch}\left(n\right)\right\}}_{n=1}^{N_{2}}$. The dataset forms a high-dimensional geometric space, and it is described by the mathematical expression of a convex hull as

(29) $$A{X_{ch}}^{T}\left(n\right)-B\leq 0,$$

where

$$\left\{\begin{array}{cc} A & ={\left[a^{\left(1\right)},\ldots ,a^{\left(p\right)},\ldots ,a^{\left(P\right)}\right]}^{T},\\ B & ={\left[b^{\left(1\right)},\ldots ,b^{\left(p\right)},\ldots ,b^{\left(P\right)}\right]}^{T},\\ 0 & ={\left[0,0,\ldots ,0\right]}^{T}. \end{array}\right.$$

Here, $a^{\left(p\right)}$ is the unit normal vector of *p*-th hyperplane, and $b^{\left(p\right)}$ is the distance of *p*-th hyperplane away from the origin point [30].

### 4.2. Entropy Weight Approach

First, the Pareto frontier represents the set of all non-dominated solutions in a multi-objective optimization problem. This implies that no single solution can optimize all objectives simultaneously. Therefore, the entropy weight approach is applied to transform the multi-objective optimization into a single-objective one [31].

When the existence of a solution, X(1), in the dataset *X* is assumed, it must satisfy the following criterion to be considered part of the Pareto front: (1) $f_{k}\left(X\left(1\right)\right)\leq f_{k}\left(X\right)$, ∀k∈{1,2,3}; (2). $f_{k^{*}}\left(X\left(1\right)\right)<f_{k^{*}}\left(X\right)$, $\exists k^{*}\in \left\{1,2,3\right\}$. Based on the criterion, the optimal set of solutions for the Pareto front from the dataset ${\left\{X_{ch}\left(n\right)\right\}}_{n=1}^{N_{2}}$ in (29) is ${\left\{X_{p}\left(n\right)\right\}}_{n=1}^{N}$.

Second, based on the definition of information entropy, the entropy weight, $w_{k}$, can be computed as

(30) $$w_{k}=\frac{1+\frac{1}{\ln N}\sum_{n=1}^{N}P_{nk}\ln P_{nk}}{\sum_{k=1}^{3}\left(1+\frac{1}{\ln N}\sum_{n=1}^{N}P_{nk}\ln P_{nk}\right)},$$

where $P_{nk}$ is the normalized proportion matrix of the objective values, which can be expressed as

$$P_{nk}=\left[\begin{array}{ccc} \frac{f_{1}\left(X_{p}\left(1\right)\right)}{\sum_{n=1}^{N}f_{1}\left(x_{p}\left(n\right)\right)} & \frac{f_{2}\left(X_{p}\left(1\right)\right)}{\sum_{n=1}^{N}f_{2}\left(X_{p}\left(n\right)\right)} & \frac{f_{3}\left(X_{p}\left(1\right)\right)}{\sum_{n=1}^{N}f_{3}\left(X_{p}\left(n\right)\right)}\\ \vdots & \vdots & \vdots \\ \frac{f_{1}\left(X_{p}\left(N\right)\right)}{\sum_{n=1}^{N}f_{1}\left(X_{p}\left(n\right)\right)} & \frac{f_{2}\left(X_{p}\left(N\right)\right)}{\sum_{n=1}^{N}f_{2}\left(X_{p}\left(n\right)\right)} & \frac{f_{3}\left(X_{p}\left(N\right)\right)}{\sum_{n=1}^{N}f_{3}\left(X_{p}\left(n\right)\right)} \end{array}\right].$$

Here, the functions $f_{1}$, $f_{2}$, and $f_{3}$ correspond to the objective functions $G_{L}$, $S_{L}$, and Cost in (28), respectively.

Finally, the three objective functions are combined into a single objective function, *f*, using entropy weights ${\left\{w_{k}\right\}}_{k=1}^{3}$ in (30). This objective function can be expressed as

(31) $$f=w_{1}\cdot |G_{L}|+w_{2}\cdot \left|S_{L}\right|+w_{3}\cdot Cost.$$

### 4.3. Optimal Scheduling Method

This subsection introduces an optimal scheduling method based on the operating zone model. The optimal solution is obtained in a visual manner in the following stages:

*(1) The first-stage optimization:*

The optimization objective of the first stage is to minimize the gas loss, steam loss, and operating costs without considering the gas holder (i.e., $G_{H}$ = 0 ${\mathrm{km}}^{3}/h$). The objective function is expressed as

(32) $$f_{L}=w_{1}\cdot \left|{G_{L}}^{\prime }\right|+w_{2}\cdot \left|S_{L}\right|+w_{3}\cdot Cost.$$

Here,

(33) $${G_{L}}^{\prime }=\left(1-r_{R}\right)\cdot G_{0}-G_{M}-G_{B}.$$

Given the current values of the total gas flow, $G_{0}$, the turbine extraction, $S_{T}$, and the safety margin, $r_{SM}$, data points from the dataset ${\left\{X_{ch}\left(n\right)\right\}}_{n=1}^{N_{2}}$ in (29) that satisfy these input conditions are selected to form a new dataset, ${\left\{X_{fs}\left(n\right)\right\}}_{n=1}^{N_{3}}$. The projection of this dataset on the $G_{M}$–$G_{B}$ plane forms a constraint region, $R_{I}$, for the decision variables $G_{M}$ and $G_{B}$. The objective value corresponding to each data point, $X_{fs}\left(n\right)$, in $R_{I}$ is calculated in (32) as ${\left\{f_{L}\left(n\right)\right\}}_{n=1}^{N_{3}}$ := $\{f_{L}\left(X_{fs}\left(n\right)\right)|n\in [1,$$N_{3}]\}$. The optimal gas allocation value, $\left(G_{M}\left(n_{1}\right),G_{B}\left(n_{1}\right)\right)$, for the first-stage optimization is obtained; its position, $n_{1}$, in the dataset ${\left\{X_{fs}\left(n\right)\right\}}_{n=1}^{N_{3}}$ is computed as

(34) $$n_{1}=\underset{n}{\arg\min}{\left\{f_{L}\left(n\right)\right\}}_{n=1}^{N_{3}}.$$

To facilitate observation, the objective values ${\left\{f_{L}\left(n\right)\right\}}_{n=1}^{N_{3}}$ are plotted as colored contour lines on the $G_{M}$–$G_{B}$ plane within the region $R_{I}$ (see e.g., Figure 8 given later in Section 5). The optimal value of $f_{L}$ corresponds to the data point with the minimum value at $G_{H}$ = 0 ${\mathrm{km}}^{3}/h$.

*(2) The second-stage optimization:*

The optimization objective of the second stage is to solve the optimization problem in (28) by considering the energy storage constraint of the gas holder based on the constraint region, $R_{I}$, and the objective values ${\left\{f_{L}\left(n\right)\right\}}_{n=1}^{N_{3}}$.

Given the current value of the gas holder volume, $V_{H}$, the constraint range for the storage gas, $G_{H}$, is calculated from (24) as $G_{H,min}\leq G_{H}\leq G_{H,max}$. Based on Equation (21), an energy storage region, $R_{II}$, on the $G_{M}$–$G_{B}$ plane can be defined as

(35) $$R_{II}=\left\{\left(G_{M},G_{B}\right)|G_{H,min}\leq \left(1-r_{R}\right)\cdot G_{0}-G_{M}-G_{B}\leq G_{H,max}\right\}.$$

If $R_{I}\cap R_{II}\neq ⌀$, the gas holder volume can store (or supply) the excess (or deficit) gas in the system, thus ensuring zero gas loss. Under known conditions of $V_{H}$, the values of $G_{H}$ are given by ${\left\{G_{H}\left(j\right)\right\}}_{j=1}^{N_{H}}$ = $G_{H,min}$: δ: $G_{H,max}$. For any given gas holder storage level, $G_{H}\left(j\right)$, a gas balance line (see e.g., the red dashed lines in Figure 9 given later in Section 5), $L_{G}$, at $G_{L}$ = 0 ${\mathrm{km}}^{3}/h$ can be obtained from (21). Using the objective values ${\left\{f_{L}\left(n\right)\right\}}_{n=1}^{N_{3}}$, the general solution to the optimization problem in (28) is determined as ${\left\{\left(G_{M}\left(n_{2}\left(j\right)\right),G_{B}\left(n_{2}\left(j\right)\right),G_{H}\left(n_{2}\left(j\right)\right)\right)\right\}}_{j=1}^{N_{H}}$. The positions ${\left\{n_{2}\left(j\right)\right\}}_{j=1}^{N_{H}}$ in the dataset ${\left\{X_{fs}\left(n\right)\right\}}_{n=1}^{N_{3}}$ are calculated as

(36) $${\left\{n_{2}\left(j\right)\right\}}_{j=1}^{N_{H}}={\left\{\underset{n}{\arg\min}{\left\{f_{L}\left(n\right)|G_{L}\left(G_{M}{\left(n\right),G}_{B}\left(n\right),G_{H}\left(j\right)\right)=0\right\}}_{n=1}^{N_{3}}\right\}}_{j=1}^{N_{H}}.$$

If $R_{I}\cap R_{II}=⌀$, it indicates that the gas holder cannot fully balance the excess or deficit gas in the system, resulting in inevitable gas losses. When there is an excess (or deficit) of gas, i.e., ${{\;G}_{L}}^{\prime }\left(n_{1}\right)>0$$\left({{\;G}_{L}}^{\prime }\left(n_{1}\right)<0\right)$, the gas holder needs to use its maximum storage (or supply) to buffer this imbalance as much as possible. Based on the optimization result $\left(G_{M}\left(n_{1}\right),G_{B}\left(n_{1}\right)\right)$ of the first stage, the optimal gas allocation value $\left(G_{M}^{*},G_{B}^{*},G_{H}^{*}\right)$ can be adjusted as

(37) $$\left(G_{M}^{*},G_{B}^{*},G_{H}^{*}\right)=\left\{\begin{array}{c} \left(G_{M}\left(n_{1}\right),G_{B}\left(n_{1}\right),G_{H,max}\right),\;\;\;\;\mathrm{if}\;{G_{L}}^{\prime }\left(n_{1}\right)>0,\\ \left(G_{M}\left(n_{1}\right),G_{B}\left(n_{1}\right),G_{H,min}\right),\;\;\;\;\mathrm{if}\;{G_{L}}^{\prime }\left(n_{1}\right)<0. \end{array}\right.$$

The optimization problem addressed in the second stage can also be solved using traditional optimization approaches, yielding the same results as the proposed method. However, the proposed method provides a visual representation of the decision-making process, which is not available in traditional approaches. This feature assists industrial plant operators in understanding, evaluating, and managing decisions related to optimization objectives. Machine learning-based optimization approaches can achieve high optimization performance when a large amount of data samples is available. However, the approaches are highly reliant on extensive data samples and have limited extrapolation properties in data-scarce scenarios. In contrast, the proposed method, grounded in mechanistic modeling, offers a good extrapolation property.

## 5. Case Studies

**Figure 2:**
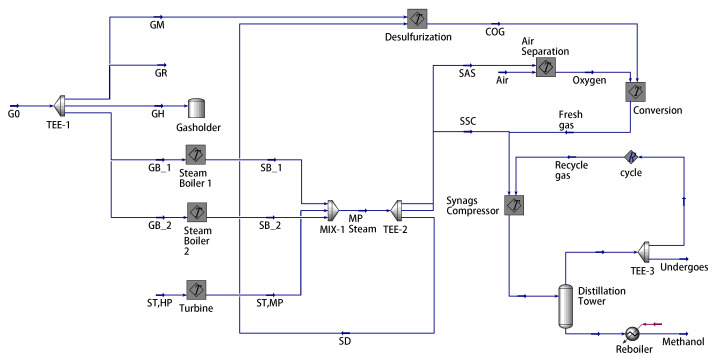
The Aspen Hysys model of the gas-to-methanol process.

**Figure 3:**
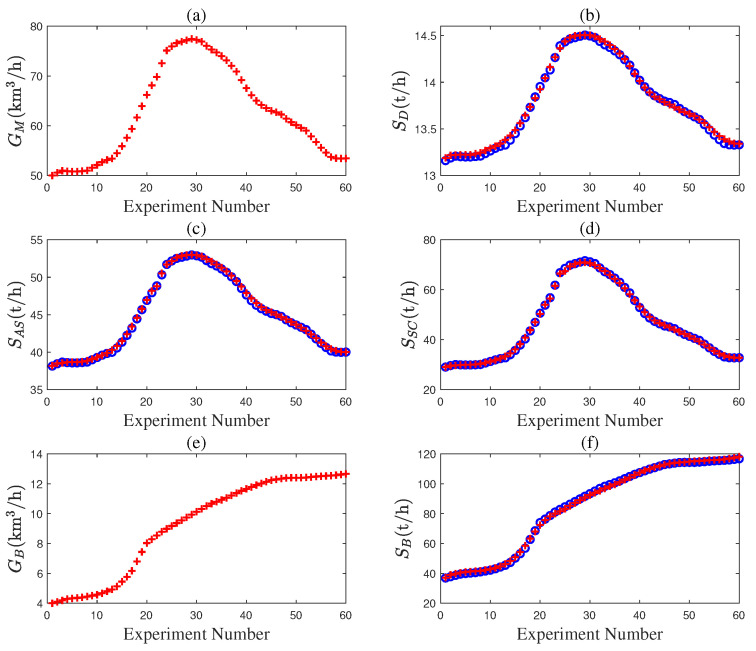
Input and output data of mechanistic models (blue circle) and Aspen Hysys model (red cross): (**a**) $G_{M}$; (**b**) $S_{D}$; (**c**) $S_{AS}$; (**d**) $S_{SC}$; (**e**) $G_{B}$; (**f**) $S_{B}$.

**Figure A1:**
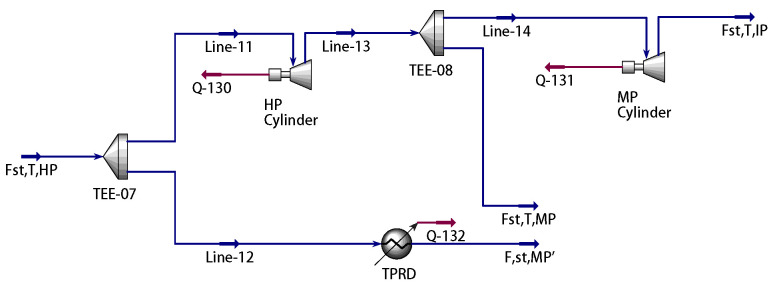
Steam turbine model based on Aspen Hysys.

**Figure A2:**
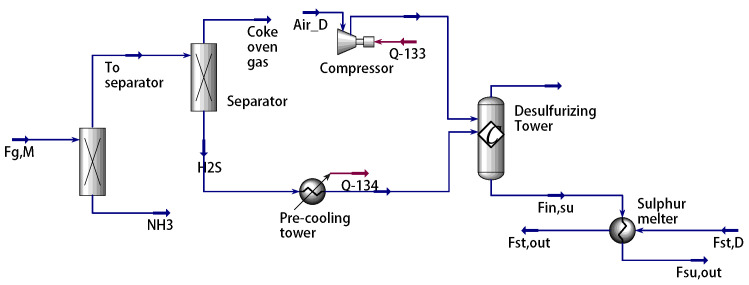
Desulfurization model based on Aspen Hysys.

**Figure A3:**
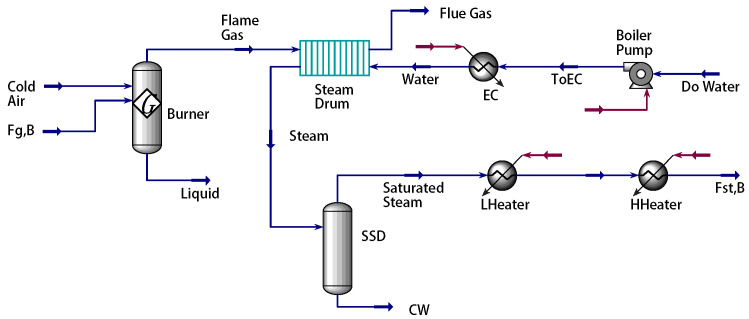
Steam boiler model based on Aspen Hysys.

**Figure A4:**
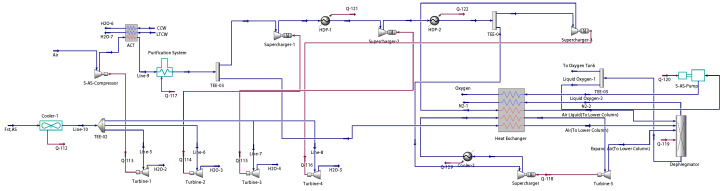
Air separation model based on Aspen Hysys.

**Figure A5:**
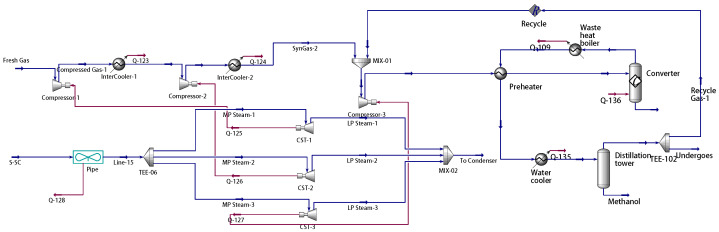
Syngas compressor model based on Aspen Hysys.

**Figure A6:**
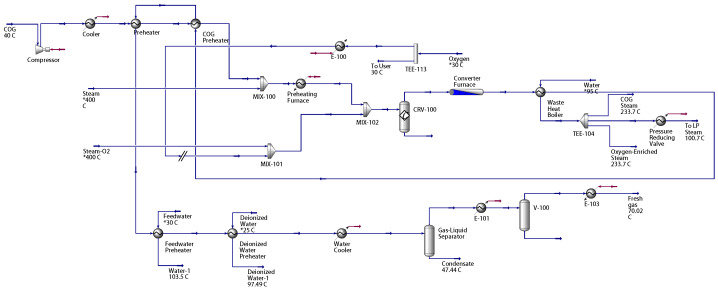
Conversion section model based on Aspen Hysys.

This section provides case studies based on Aspen Hysys to support and validate the proposed method. The mechanistic models of the desulfurization, air separation, syngas compressor, and steam boiler are given in (17). A simulation model of the gas-to-methanol process in Figure 2 is set up using Aspen Hysys V11 (Bedford, MA, USA). Figure A1, Figure A2, Figure A3, Figure A4, Figure A5 and Figure A6 depict the Aspen Hysys models of each subsystem in this process [32]. Given the nominal values of the measurable variables in Table 1 and the known parameters in Table 2, the unknown parameters $\theta_{D}$, $\theta_{AS}$, $\theta_{SC}$, and $\theta_{B}$ in (16) are estimated using data from the Aspen Hysys model as shown in Figure 3. The estimation results are shown in Table 3. The output data from the mechanistic models is overlapped with the counterparts from the Aspen Hysys model in Figure 3b–f. In particular, the numerical results in Figure 3c illustrate that the ideal gas assumption in the air separation model is reasonable. Hence, the developed models are quite accurate and can be applied to subsequent optimal scheduling tasks.

**Figure 4:**
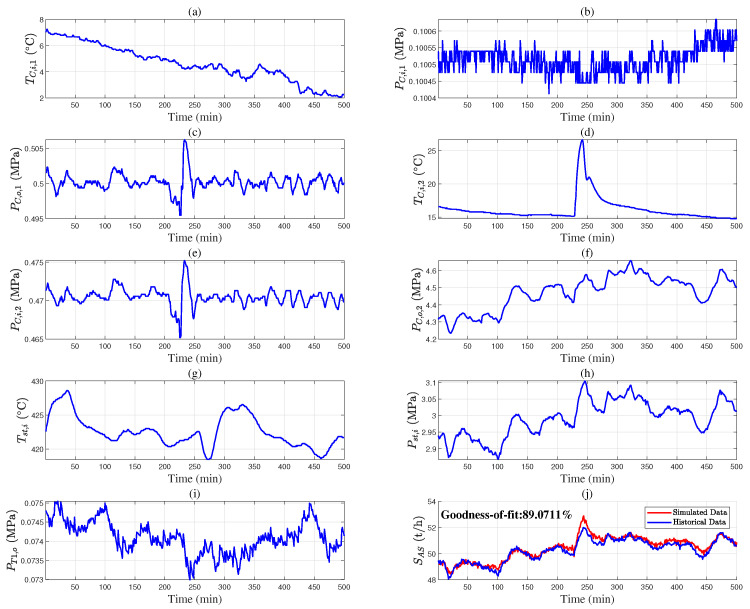
Parameter estimation of air separation models based on historical data: (**a**) $T_{C,i,1}$, (**b**) $P_{C,i,1}$, (**c**) $P_{C,o,1}$, (**d**) $T_{C,i,2}$, (**e**) $P_{C,i,2}$, (**f**) $P_{C,o,2}$, (**g**) $T_{st,i}$, (**h**) $P_{st,i}$, (**i**) $P_{T1,o}$, and (**j**) ${\hat{S}}_{AS}$ (red solid line) and $S_{AS}$ (blue solid line).

**Figure 5:**
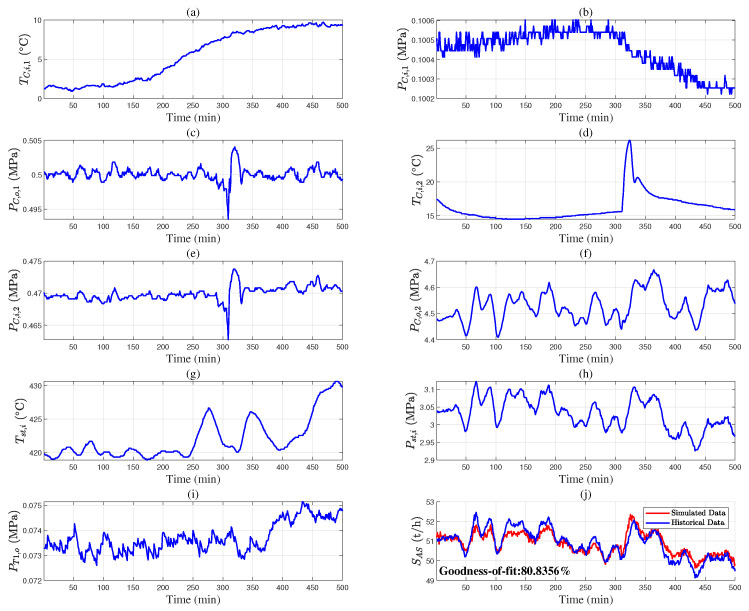
Parameter validation of air separation models based on historical data: (**a**) $T_{C,i,1}$, (**b**) $P_{C,i,1}$, (**c**) $P_{C,o,1}$, (**d**) $T_{C,i,2}$, (**e**) $P_{C,i,2}$, (**f**) $P_{C,o,2}$, (**g**) $T_{st,i}$, (**h**) $P_{st,i}$, (**i**) $P_{T1,o}$, and (**j**) ${\hat{S}}_{AS}$ (red solid line) and $S_{AS}$ (blue solid line).

**Figure 6:**
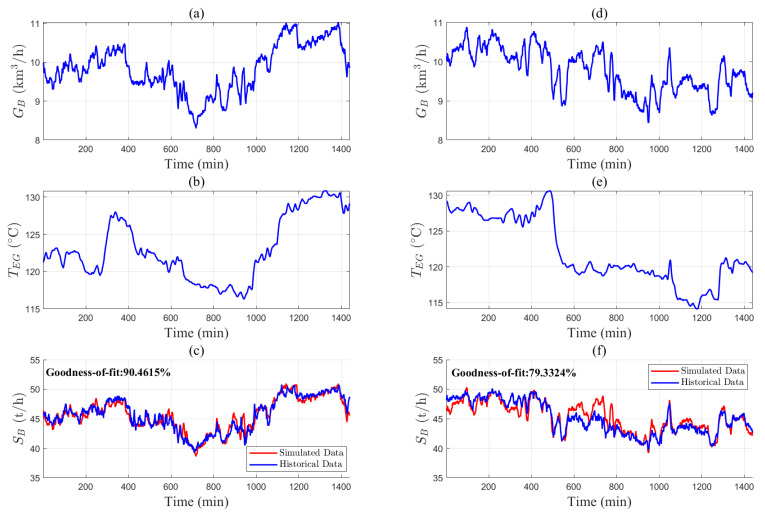
Parameter estimation of steam boiler models based on historical data: (**a**) $G_{B}$, (**b**) $T_{EG}$, and (**c**) ${\hat{S}}_{B}$ (red solid line) and $S_{B}$ (blue solid line); parameter validation of steam boiler models based on historical data: (**d**) $G_{B}$, (**e**) $T_{EG}$, and (**f**) ${\hat{S}}_{B}$ (red solid line) and $S_{B}$ (blue solid line).

The industrial data are used to validate the accuracy of the mechanistic models. Figure 4, Figure 5 and Figure 6 provide the industrial data segments of the air separation and steam boiler models. The unknown model parameters are estimated by maximizing the goodness of fit between the measured output *y* and the simulated output $\hat{y}$ from the mechanistic model;

(38) $$\hat{\theta }=\underset{\theta }{\arg\max}\left(1-\sum_{t=1}^{T}\frac{{\left(y\left(t\right)-\hat{y}\left(t,\theta \right)\right)}^{2}}{{\left(y\left(t\right)-\frac{1}{T}\sum_{t=1}^{T}y\left(t\right)\right)}^{2}}\right).$$

The model parameters are calculated as $\theta_{AS,1}=2.16$, $\theta_{AS,2}=4.31$, $\theta_{AS,3}=23.96$, $\theta_{B,1}=9.92$, and $\theta_{B,2}=0.0058$ using Equation (38). Figure 4j and Figure 6c show the fitting results of the models. The data segments in Figure 5 and Figure 6d–f are used for model validation, with goodness of fit values of 80.84% and 79.33%, respectively. These results indicate that the air separation and steam boiler models in Equations (7) and (15) are valid.

**Figure 7:**
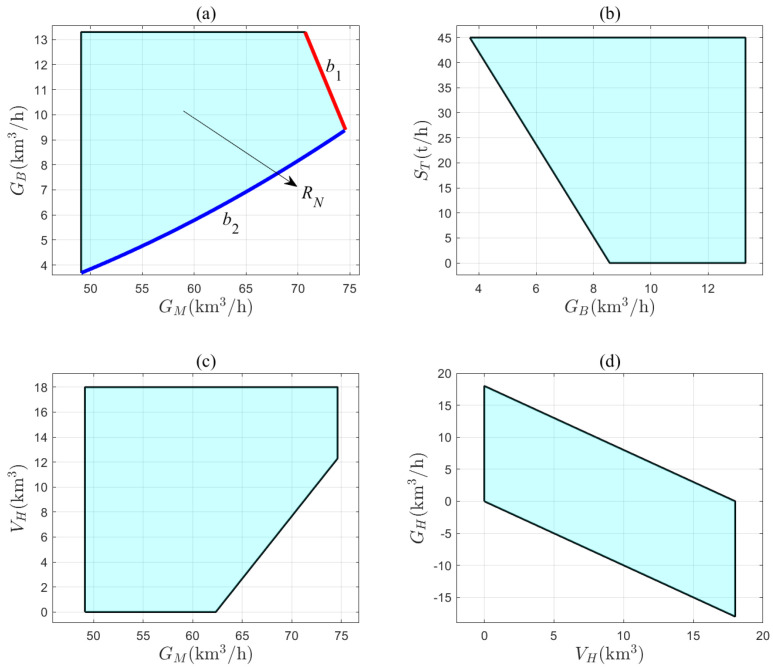
The projection regions of the operating zone model on (**a**) $G_{M}$–$G_{B}$; (**b**) $G_{B}$-$S_{T}$; (**c**) $G_{M}$-$V_{H}$; (**d**) $V_{H}$-$G_{H}$ planes.

To establish an operating zone model, the variation ranges of the variables $G_{0}$, $S_{T}$, $V_{H}$, $G_{M}$, and $G_{B}$ in (20) are given in Table 4. The dataset for constructing the operating zone model is obtained as ${\left\{X_{ch}\left(n\right)\right\}}_{n=1}^{333220}$, which is described in the form of a convex hull in (29). The operating zone model shows its projection regions on the two-dimensional planes in Figure 7. Each boundary of these regions has a corresponding physical meaning. For example, Figure 7a shows the projection region $R_{N}$ of the operating zone model on the $G_{M}$–$G_{B}$ plane. The physical meanings of the boundaries $b_{1}$ and $b_{2}$ are elaborated here.

(1) The boundary $b_{1}$ corresponds to the operating conditions for $G_{0}$ = $G_{0,max}$, $G_{H}$ = $G_{H,min}$, and $G_{L}$ = 0 ${\mathrm{km}}^{3}/h$. The mathematical expression for this boundary can be obtained from (21) as
  (39) $$G_{B}=\left(1-r_{R}\right)\cdot G_{0,max}-G_{M}-G_{H,min}.$$
  The total gas supply, $G_{0}$, of the coke oven is maximized, and the full-capacity gas holder supplies all the gas to the system. As the demand for gas, $G_{M}$, increases, the system can only maintain the balance between gas supply and demand by reducing the allocation of gas, $G_{B}$, to the boiler.
(2) The boundary $b_{2}$ corresponds to the operating condition for $S_{T}$ = $S_{T,max}$, $S_{L}$ = 0 t/h, and $r_{SM}$ = 0 %. The mathematical description of this boundary is given by (25) as
  (40) $$G_{B}={f_{B}}^{-1}\left(f_{D}\left(G_{M}\right)+f_{AS}\left(G_{M}\right)+f_{SC}\left(G_{M}\right)-S_{T,max}\right),$$
  where ${f_{B}}^{-1}$ denotes the inverse function of $f_{B}$. The system has two sources of steam supply: the steam extraction, $S_{T}$, from the turbine and the steam generation, $S_{B}$, from the boiler. When $S_{T}$ reaches its maximum value and the steam backup in the steam subsystem is fully utilized, the only way to maintain the balance between steam supply and demand as the gas demand, $G_{M}$, increases is to burn more gas, $G_{B}$, in the boiler to produce enough $S_{B}$.

The optimization problem in (28) can now be solved. The total gas flow, $G_{0}$, is 110 ${\mathrm{km}}^{3}/h$, the turbine extraction, $S_{T}$, is 35 t/h, the gas holder volume, $V_{H}$, is 8 ${\mathrm{km}}^{3}$, and the safety margin, $r_{SM}$, is 10% for steam backup. The following price information is given: 234 CNY/kg for $c_{st,T}$, 500 $\mathrm{CNY}/m^{3}$ for $c_{gas}$, and 195 CNY/kg for $c_{st,MP}$. Based on the dataset ${\left\{X_{ch}\left(n\right)\right\}}_{n=1}^{333220}$, the entropy weights are calculated using (30), with $w_{1}=0.5430$, $w_{2}=0.2478$, and $w_{3}=0.2092$.

**Figure 8:**
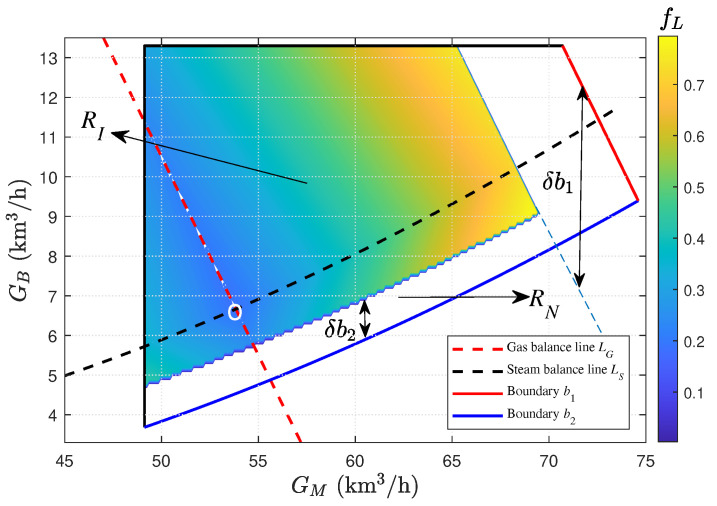
The first-stage optimization based on the operating zone model.

In the first stage, the dataset ${\left\{X_{fs}\left(n\right)\right\}}_{n=1}^{237560}$ that meets the input conditions $G_{0}$ = 110 ${\mathrm{km}}^{3}/h$ and $S_{T}$ = 35 t/h is obtained, and a constraint region $R_{I}$ shown in Figure 8 is the projection of this dataset onto the $G_{M}$–$G_{B}$ plane. From (39) and (40), it is understood that the physical meanings of the boundaries $b_{1}$ and $b_{2}$ of the projection region $R_{N}$ are limited by $G_{0,max}$ and $S_{T,max}$, respectively. If $G_{0}$ and $S_{T}$ are given, the operating conditions of $b_{1}$ and $b_{2}$ cannot reach $G_{0,max}$ and $S_{T,max}$. Therefore, the displacements of the boundaries from $R_{N}$ to $R_{I}$ along the vertical axis can be expressed as $\delta_{b_{1}}=\left(1-r_{R}\right)\cdot \left(G_{0}-G_{0,max}\right)$ and $\delta_{b_{2}}=\frac{{S_{T,max}-S}_{T}}{\theta_{B,1}}$. Here, $\delta_{b_{1}}\leq 0$ and $\delta_{b_{2}}\geq 0$ indicate that $b_{1}$ and $b_{2}$ move downward and upward, respectively, causing the projection region from $R_{N}$ to $R_{I}$ to shrink inward. Substituting the dataset ${\left\{X_{fs}\left(n\right)\right\}}_{n=1}^{237560}$ into (32) yields the objective values ${\left\{f_{L}\left(n\right)\right\}}_{n=1}^{237560}$, which are plotted as a contour map in Figure 8. The color distribution indicates that, as the values in the right color bar increase from low to high, the objective values expand from the white circle. In (34), the optimal point (white circle) for the objective $f_{L}$ is calculated as $\left(G_{M}^{*}\left(31890\right),G_{B}^{*}\left(31890\right)\right)$ = (53.84,6.66). The gas balance line, $L_{G}$ (red dashed line), and the steam balance line, $L_{S}$ (black dashed line), under the conditions of ${G_{L}}^{\prime }$ = 0 ${\mathrm{km}}^{3}/h$ and $S_{L}$ = 0 t/h, respectively, are plotted from (33) and (25). Their intersection is the optimal point obtained in (34), which indicates that, at this point, both the gas and steam subsystems can simultaneously achieve the zero-loss objective.

**Figure 9:**
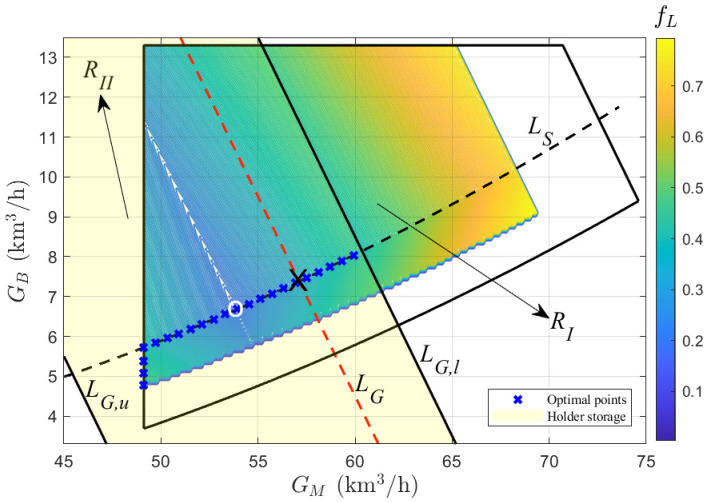
The second-stage optimization based on the operating zone model.

In the second stage, based on the gas holder volume $V_{H}$ of 8 ${\mathrm{km}}^{3}$, the gas storage $G_{H}\in \left[-8,10\right]\;{\mathrm{km}}^{3}/h$ is calculated in (24). An energy storage region, $R_{II}$ (yellow region), in (35) is given in Figure 9, where the upper limit, $L_{G,u}$, and lower limit, $L_{G,l}$, of $R_{II}$ correspond to the operating conditions for $G_{H}$ = 10 ${\mathrm{km}}^{3}/h$ and $G_{H}$ = −8 ${\mathrm{km}}^{3}/h$, respectively. Since $R_{I}\cap R_{II}\neq ⌀$, the optimal point can be calculated using (36) at different storage levels of the gas holder and is marked with a blue cross in the intersecting region of $R_{I}$ and $R_{II}$. If the value of $G_{H}$ is chosen to be −4 ${\mathrm{km}}^{3}/h$, the gas balance line $L_{G}$ (red dashed line) can be obtained using (21), as shown in Figure 9. The intersection of $L_{G}$ and $L_{S}$ represents the optimal gas allocation value, $\left(G_{M}^{*},G_{B}^{*}\right)$.

The scheduling schemes for the first and second stages are presented in Table 5. To validate the feasibility of the energy allocation, $G_{M}^{*}$ and $G_{B}^{*}$ are fed into the Aspen Hysys model in Figure 2. The simulation results from the Aspen Hysys model are provided in Table 5. To assess the accuracy of the optimization results, an error percentage, *E*, is defined as

$$E=\frac{\left|\mathrm{real}\;\mathrm{value}-\mathrm{calculated}\;\mathrm{value}\right|}{\mathrm{nominal}\;\mathrm{value}}\times 100\%.$$

Here, the real values are obtained from Aspen Hysys, the calculated values are provided via the proposed method, and the nominal values are given in the sixth column of Table 5, based on the process operation manual. In general, the acceptable criterion for *E* should be less than 5%. All error percentages in the last two columns of Table 5 are less than 2%, showing that the calculated values are consistent with the real values. To evaluate the parameter sensitivity, a positive perturbation of 1% is introduced to all parameters in Table 3. The experiment was then repeated with the updated parameter values in Table 6. The optimized results are summarized in Table 7. These results remain consistent with those in the second and third columns of Table 5, demonstrating the credibility of the proposed method.

## 6. Conclusions

This paper has proposed an optimal scheduling method using operating zone models and entropy weights for an energy system in a gas-to-methanol process. The first step was to develop mechanistic models for major production facilities, including desulfurization, air separation, syngas compression, and steam boilers, based on their physical mechanisms. The unknown model parameters were then estimated using historical data through the genetic algorithm. A multi-objective optimization problem was formulated, with the objectives of minimizing gas loss, steam loss, and operating costs. Operation constraints were mainly from the equipment capacities, energy balance, and energy coupling relationships. The entropy weight approach was then employed to convert this problem into a single-objective optimization problem. The second step was to solve the optimization problem based on the operating zone model. Through a projection of the operating zone model onto the decision variable plane, the optimal scheduling solution was obtained in a visual manner with contour lines and auxiliary lines. Case studies based on Aspen Hysys illustrated the effectiveness of the proposed method.

In the case studies, the mechanistic models for air separation and steam boilers were validated using industrial data. The remaining models have yet to be validated. In future work, the models for desulfurization and syngas compressors will be validated using industrial data. The validated models will then be integrated into the optimization framework for scheduling in a real industrial setting.

## Figures and Tables

**Table 1:** Nominal values and operation ranges of measurable variables.

Variable	Nominal Value	Operation Range	Variable	Nominal Value	Operation Range
$T_{su,i}$	30 °C	20–35 °C	$T_{su,o}$	83.5 °C	80–115 °C
$T_{st,i}$	435.9 °C	400–450 °C	$T_{st,o}$	371 °C	365–394 °C
$T_{C,i,1}$	20 °C	5–40 °C	$T_{C,i,2}$	11 °C	11–12 °C
$P_{C,i,1}$	0.1 MPa	0.07–1 MPa	$P_{C,o,1}$	0.5 MPa	0.45–0.55 MPa
$P_{C,i,2}$	0.5 MPa	0.45–0.55 MPa	$P_{C,o,2}$	4 MPa	-
$P_{st,i}$	3.43 MPa	≥3.0 MPa	$P_{T1,o}$	0.07 MPa	0–0.3 MPa
$T_{SC,i,s}$	40 °C	-	$T_{SC,o,1}$	88 °C	85–105 °C
$T_{SC,o,2}$	84 °C	85–105 °C	$T_{SC,o,3}$	48 °C	45–60 °C
$P_{SC,i,1}$	2.1 MPa	-	$P_{SC,o,1}$	3.47 MPa	-
$P_{SC,i,2}$	3.47 MPa	-	$P_{SC,o,2}$	5.5 MPa	-
$P_{SC,i,3}$	5.5 MPa	-	$P_{SC,o,3}$	6 MPa	-
$P_{T2,o}$	0.018 MPa	0–0.3 MPa	$T_{EG}$	120 °C	≤170 °C
$T_{CA}$	25 °C	20–30 °C	-	-	-

**Table 2:** Known parameters of models.

Parameter	Value	Parameter	Value
*R*	0.287 kJ/kg·K	$\eta_{I,1}$	80%
$\eta_{I,2}$	75%	$r_{O}$	0.22
$\eta_{T1}$	75%	$k_{T1}$	1.37
$R_{u}$	8.314 kJ/kg·K	$M_{1}$,$M_{2}$	11.232 g/mol
$M_{3}$	10.171 g/mol	$\eta_{P,1}$	80%
$\eta_{P,2}$	75%	$\eta_{P,3}$	78%
$r_{f}$	1.42	$C_{1}$	0.14
$Q_{B}$	17,900 $\mathrm{kJ}/m^{3}$	$r_{R}$	0.45

**Table 3:** Unknown parameters of models.

Parameter	Value	Parameter	Value	Parameter	Value
$\theta_{D,1}$	0.059	$\theta_{D,2}$	12.99	$\theta_{AS,1}$	2.83
$\theta_{AS,2}$	2.65	$\theta_{AS,3}$	11.15	$\theta_{SC,1}$	0.84
$\theta_{SC,2}$	0.79	$\theta_{SC,3}$	2.87	$\theta_{SC,4}$	−72
$\theta_{B,1}$	9.81	$\theta_{B,2}$	6.2 × $10^{-3}$	-	-

**Table 4:** Operation ranges for input and decision variables.

Variable	Operation Range
$G_{0}$	100–120 ${\mathrm{km}}^{3}/h$
$S_{T}$	0–45 t/h
$V_{H}$	0–18 ${\mathrm{km}}^{3}/h$
$G_{M}$	49.12–78.59 ${\mathrm{km}}^{3}/h$
$G_{B}$	3.3–13.3 ${\mathrm{km}}^{3}/h$

**Table 5:** Validation of the optimal scheduling results.

Variable	The Proposed Method		Aspen HYSYS Model		Error Percentage		
	Stage 1	Stage 2	Stage 1	Stage 2	Nominal Value	Stage 1	Stage 2
$G_{H}^{*}\;\left({\mathrm{km}}^{3}/h\right)$	0	−4	0	−4	-		
$G_{M}^{*}\;\left({\mathrm{km}}^{3}/h\right)$	53.84	57.12	53.84	57.12	-		
$G_{B}^{*}\;\left({\mathrm{km}}^{3}/h\right)$	6.66	7.38	6.66	7.38	-		
$G_{L}^{*}\;\left({\mathrm{km}}^{3}/h\right)$	0	0	0	0	-		
$S_{D}^{*}\;\left(t/h\right)$	13.35	13.53	13.36	13.55	15.8	0.06%	0.13%
$S_{AS}^{*}\;\left(t/h\right)$	40.22	41.99	40.19	42.17	42.7	0.07%	0.42%
$S_{SC}^{*}\;\left(t/h\right)$	33.23	37.26	33.25	37.48	31.3	0.06%	0.7%
$S_{B}^{*}\;\left(t/h\right)$	61.44	68.04	61.57	68.85	65.6	0.19%	1.2%
$S_{L}^{*}\;\left(t/h\right)$	0	0	0.11	0.27	28.44	0.39%	0.95%

**Table 6:** Unknown parameters of models based on parameter sensitivity.

Parameter	Value	Parameter	Value	Parameter	Value
$\theta_{D,1}$	0.0596	$\theta_{D,2}$	13.12	$\theta_{AS,1}$	2.86
$\theta_{AS,2}$	2.677	$\theta_{AS,3}$	11.26	$\theta_{SC,1}$	0.848
$\theta_{SC,2}$	0.798	$\theta_{SC,3}$	2.899	$\theta_{SC,4}$	−72.72
$\theta_{B,1}$	9.91	$\theta_{B,2}$	6.26 × $10^{-3}$	-	-

**Table 7:** Optimal scheduling results based on parameter sensitivity.

Variable	The Proposed Method	
	Stage 1	Stage 2
$G_{H}^{*}\;\left({\mathrm{km}}^{3}/h\right)$	0	−4
$G_{M}^{*}\;\left({\mathrm{km}}^{3}/h\right)$	53.74	57.01
$G_{B}^{*}\;\left({\mathrm{km}}^{3}/h\right)$	6.76	7.48
$G_{L}^{*}\;\left({\mathrm{km}}^{3}/h\right)$	0.09	0.01
$S_{D}^{*}\;\left(t/h\right)$	13.47	13.64
$S_{AS}^{*}\;\left(t/h\right)$	40.53	42.35
$S_{SC}^{*}\;\left(t/h\right)$	34.14	38.20
$S_{B}^{*}\;\left(t/h\right)$	62.95	69.66
$S_{L}^{*}\;\left(t/h\right)$	0	0

## Data Availability

All data are presented in the main text.

## Nomenclature

$C_{1}$	coefficient of the polynomial.
$C_{2}$	coefficient of the polynomial.
$C_{3}$	coefficient of the polynomial.
$C_{st}$	specific heat capacity of steam at constant pressure, kJ/(kg · °C).
$C_{su}$	specific heat capacity of sulfur at constant pressure, kJ/(kg · °C).
$F_{A}$	inlet flow of air, ${\mathrm{km}}^{3}/h$.
$F_{su}$	inlet flow of sulfur, t/h.
$G_{0}$	total gas flow, ${\mathrm{km}}^{3}/h$.
$G_{B}$	gas combustion flow of boiler, ${\mathrm{km}}^{3}/h$.
$G_{H}$	gas storage flow of gas holder, ${\mathrm{km}}^{3}/h$.
$G_{L}$	loss flow of gas subsystem, ${\mathrm{km}}^{3}/h$.
$G_{M}$	gas conversion flow of methanol production, ${\mathrm{km}}^{3}/h$.
$G_{R}$	recycled gas flow of coke oven, ${\mathrm{km}}^{3}/h$.
$H_{S}$	enthalpy value of steam, kJ/kg.
$H_{FW}$	enthalpy value of feed water, kJ/kg.
$M_{s}$	molar mass of inlet gas of *s*-th syngas compressor, g/mol.
$P_{C,i,s}$	air inlet pressure of *s*-th compressor, MPa.
$P_{C,o,s}$	air outlet pressure of *s*-th compressor, MPa.
$P_{SC,i,s}$	inlet pressure of *s*-th syngas compressor, MPa.
$P_{SC,o,s}$	outlet pressure of *s*-th syngas compressor, MPa.
$P_{T1,o}$	steam outlet pressure of air separation turbine, MPa.
$P_{T2,o}$	steam outlet pressure of syngas compressor tubine, MPa.
$P_{st,i}$	inlet pressure of steam, MPa.
$Q_{B}$	total heat value of fuel, $\mathrm{kJ}/m^{3}$.
$Q_{st}$	heat released from steam, kJ/h.
$Q_{su}$	heat of sulfur absorption, kJ/h.
*R*	gas constant for air, kJ/kg·K.
$R_{u}$	universal gas constant, kJ/kg·K.
$S_{B}$	steam generation flow from boiler, t/h.
$S_{D}$	steam consumption flow for desulfurization, t/h.
$S_{L}$	loss flow of steam subsystem, t/h.
$S_{T}$	steam extraction flow from turbine, t/h.
$S_{AS}$	steam consumption flow for air separation, t/h.
$S_{SC}$	steam consumption flow for syngas compressor, t/h.
$T_{C,i,s}$	air inlet temperature of *s*-th compressor, °C.
$T_{CA}$	cold air temperature, °C.
$T_{EG}$	exhaust gas temperature, °C.
$T_{SC,i,s}$	inlet temperature of *s*-th syngas compressor, °C.
$T_{SC,o,s}$	outlet temperature of *s*-th syngas compressor, °C.
$T_{T1,o}$	exhaust temperature of air separation turbine, °C.
$T_{st,i}$	inlet temperature of steam, °C.
$T_{st,o}$	outlet temperature of steam, °C.
$T_{su,i}$	inlet temperature of sulfur, °C.
$T_{su,o}$	outlet temperature of sulfur, °C
$V_{H}$	volume of gas holder, ${\mathrm{km}}^{3}$.
$W_{C,s}$	power consumption of *s*-th compressor, MW.
$W_{SC,s}$	power consumption of *s*-th syngas compressor, MW.
$W_{T1}$	power output of air separation turbine, MW.
$W_{T2}$	power output of syngas compressor turbine, MW.
$X_{A}$	molar fraction of air, %.
$X_{N}$	molar fraction of nitrogen, %.
$X_{O}$	molar fraction of oxygen, %.
$\alpha_{EG}$	excess air coefficient for exhaust gas.
$\eta_{I,s}$	isentropic efficiency of *s*-th compressor, %.
$\eta_{P,s}$	polytropic efficiency of *s*-th syngas compressor, %.
$\eta_{T1}$	isentropic efficiency of air separation turbine, %.
$\eta_{T2}$	isentropic efficiency of syngas compressor turbine, %.
$\rho_{A,s}$	relative density of inlet air of *s*-th compressor.
$\rho_{SC,s}$	relative density of inlet gas of *s*-th syngas compressor.
$k_{B,1}$	calculation coefficient.
$k_{B,2}$	calculation coefficient.
$k_{T1}$	adiabatic index of steam of air separation turbine.
$k_{T2}$	adiabatic index of steam of syngas compressor tubine.
$m_{s}$	polytropic index of *s*-th syngas compressor.
$q_{1}$	effective utilization rate of boiler, %.
$q_{2}$	exhaust loss of boiler, %.
$q_{3}$	heat loss due to incomplete combustion of gases, %.
$q_{4}$	heat loss due to incomplete combustion of solid fuels, %.
$r_{O}$	oxygen to gas $G_{M}$ flow rate ratio.
$r_{R}$	recycled gas to total gas $G_{0}$ flow rate ratio.
$r_{f}$	fresh gas to gas $G_{M}$ flow rate ratio.

## Appendix A

The simulation models of the gas-to-methanol process were established using Aspen Hysys V11, as shown in Figure A1, Figure A2, Figure A3, Figure A4, Figure A5 and Figure A6. The property packages selected for methanol and steam were Peng–Robinson and ASME Steam. The key details about the Aspen Hysys model parameter settings are provided in Table 1, Table 2 and Table A1.

**Table A1:** Key details about Aspen Hysys model parameter settings.

Process/Fluid	Equipment	Parameter Settings
Coke oven gas	Components	Oxygen: 0.8%
		Hydrogen: 53.81%
		Nitrogen: 4.91%
		Methane: 26.57%
		Ethylene: 3%
		CO: 7.5%
		CO_2_: 3.14%
Medium-pressure steam	Components	H_2_O: 100%
Air	Components	Oxygen: 21%
		Nitrogen: 79%
Steam boiler	Heat exchanger	UA: $1.027\times 10^{5}$ $\mathrm{kJ}/{(}^{\circ }C\cdot h)$
		Tube delta P: 2350 kPa
	Gibbs reactor	Maximum iterations: 100
		Tolerance: $1\times 10^{-7}$
Air separation	Distillation	Theoretical plates: 30
		Feed plate: 15
		Top pressure: 340 (kPa)
		Bottom pressure: 350 (kPa)
		Reflux ratio: 0.5
	LNG exchanger	Rating method: simple-weighted
		Tolerance: $1\times 10^{-4}$
		Maximum iterations: 25
Syngas compressor	Distillation	Theoretical plates: 15;
		Feed plate: 9
		Top pressure: 1640 (kPa)
		Bottom pressure: 1883 (kPa)
		Reflux ratio: 2
		Comp fraction: 0.99
	Conversion reactor	Feed temperature: 100 °C
		Delta P: 200 kPa
		Reaction 1: ${\mathrm{CO}}_{2}+3H_{2}↔{\mathrm{CH}}_{3}\mathrm{OH}+H_{2}O$
		Conversion (%): $C_{0}=10$
		Reaction 2: $\mathrm{CO}+2H_{2}↔{\mathrm{CH}}_{3}\mathrm{OH}$
		Conversion (%): $C_{0}=25$
	Recycle	PSD properties: $1\times 10^{-3}$;
		Transfer direction: forwards
		Maximum iterations: 100
		Flash type: PT flash
		Mode: simultaneous
		Acceleration: dominant eigenvalue
Conversion section	Conversion reactor	Feed temperature: 471.1 °C
		Reaction 1: $2H_{2}+O_{2}↔2H_{2}O$
		Conversion (%): $C_{0}=66.56$
		Reaction 2: $2{\mathrm{CH}}_{4}+O_{2}↔2\mathrm{CO}+4H_{2}$
		Conversion (%): $C_{0}=24.55$
		Reaction 3: $2\mathrm{CO}+O_{2}↔2{\mathrm{CO}}_{2}$
		Conversion (%): $C_{0}=8.89$
	Plug flow reactor	Delta P: 100 kPa
		Number of segments: 20
		Minimum step fraction: $1\times 10^{-6}$
		Particle diameter: 10 mm
		Solid density: 1750 kg/m^3^
		Length: 3 m
		Diameter: 2 m
		Number of tubes: 2500
		Void fraction: 50%
		Reaction 1: $\mathrm{CO}+H_{2}O↔{\mathrm{CO}}_{2}+H_{2}$
		Reaction 2: ${\mathrm{CH}}_{4}+H_{2}O↔\mathrm{CO}+3H_{2}$
		Forward reaction: $A=2.8\times 10^{3}$$E=2.3\times 10^{5}$
		Reverse reaction: $A^{\prime }=2.5$; $B^{\prime }=-2.3\times 10^{4}$;
		$C^{\prime }=7.2$; $D^{\prime }=-2.9\times 10^{-3}$
		Reaction 3: ${\mathrm{CH}}_{4}+2H_{2}O↔{\mathrm{CO}}_{2}+4H_{2}$;
		Forward reaction: A=1; $E=2.7\times 10^{5}$
		Reverse reaction: $A^{\prime }=-5$; $B^{\prime }=-1.8\times 10^{4}$
		$C^{\prime }=8.2$; $D^{\prime }=-2.8\times 10^{-3}$
		Reaction 4: ${\mathrm{CH}}_{4}+{\mathrm{CO}}_{2}↔2\mathrm{CO}+2H_{2}$;
		Forward reaction: $A=2.9\times 10^{-7}$; $E=2.3\times 10^{5}$
		Reverse reaction: $A^{\prime }=15$; $B^{\prime }=-2.8\times 10^{4}$;
		$C^{\prime }=6.2$; $D^{\prime }=-3.1\times 10^{-3}$
		Reaction 5: $C_{2}H_{4}+2H_{2}O↔2\mathrm{CO}+4H_{2}$;
		Forward reaction: $A=4.7\times 10^{6}$; $E=2.3\times 10^{5}$
		Reverse reaction: $A^{\prime }=2.5$; $B^{\prime }=-2.3\times 10^{4}$
		$C^{\prime }=7.2$; $D^{\prime }=-2.9\times 10^{-3}$
